# Potential Mechanism of Tibetan Medicine Liuwei Muxiang Pills against Colorectal Cancer: Network Pharmacology and Bioinformatics Analyses

**DOI:** 10.3390/ph17040429

**Published:** 2024-03-27

**Authors:** Shaochong Qi, Xinyu Liang, Zijing Wang, Haoran Jin, Liqun Zou, Jinlin Yang

**Affiliations:** 1 Department of Gastroenterology and Hepatology, West China Hospital, Sichuan University, Chengdu 610041, China; victorqiqsc@163.com (S.Q.); zijingwang@wchscu.cn (Z.W.); jin_haoran98@163.com (H.J.); 2 Sichuan University-Oxford University Huaxi Joint Center for Gastrointestinal Cancer, Frontiers Science Center for Disease-Related Molecular Network, West China Hospital, Sichuan University, Chengdu 610041, China; 3 Department of Medical Oncology, West China Hospital, Sichuan University, Chengdu 610041, China; rainxl@foxmail.com (X.L.); zouliqun1971@163.com (L.Z.)

**Keywords:** colorectal cancer, Liuwei Muxiang pills, network pharmacology, bioinformatics, traditional Tibetan medicine

## Abstract

This study aimed to explore the mechanism through which Tibetan medicine Liuwei Muxiang (LWMX) pills acts against colorectal cancer (CRC). We firstly retrieved the active ingredients and the correlated targets of LWMX pills from public databases. The CRC-related targets were determined through bioinformatic analysis of a public CRC dataset. By computing the intersection of the drug-specific and disease-related targets, LWMX pill–CRC interaction networks were constructed using the protein–protein interaction (PPI) method and functional enrichment analysis. Subsequently, we determined the hub genes using machine learning tools and further verified their critical roles in CRC treatment via immune infiltration analysis and molecular docking studies. We identified 81 active ingredients in LWMX pills with 614 correlated targets, 1877 differentially expressed genes, and 9534 coexpression module genes related to CRC. A total of 5 target hub genes were identified among the 108 intersecting genes using machine learning algorithms. The immune infiltration analysis results suggested that LWMX pills could affect the CRC immune infiltration microenvironment by regulating the expression of the target hub genes. Finally, the molecular docking outcomes revealed stable binding affinity between all target hub proteins and the primary active ingredients of LWMX pills. Our findings illustrate the anti-CRC potential and the mechanism of action of LWMX pills and provide novel insights into multitarget medication for CRC treatment.

## 1. Introduction

According to global cancer statistics, colorectal cancer (CRC) ranks third in incidence and second in mortality rate among all kinds of malignant tumors [1]. It is a critical cancer that imposes a severe public health burden worldwide. Surgical resection, chemoradiotherapy, and molecular-targeted therapy are the principal treatment strategies for patients with CRC at present [2]. However, the accompanying adverse effects, limited survival improvement, susceptibility to drug resistance, and heavy economic burden of these treatment schemes indicate that the current clinical treatment status of CRC is still not optimal [3]. Therefore, there is an urgent need to explore and develop new candidate drugs for CRC with reliable potency, minimal toxicity, and a desirable cost-effectiveness.

Traditional Chinese medicine has been utilized in anticancer therapy for a long time. Currently, a growing number of clinical trials have shown that traditional Chinese medicine is helpful for the prevention and treatment of multiple kinds of tumors. For instance, in a recent randomized controlled trial of non-small-cell lung cancer, patients who had received oral Shenlingcao liquid combined with conventional chemotherapy declared better quality of life and greater improvements in cancer-related symptoms after radical resection in comparison with those who had received conventional chemotherapy alone [4]. In terms of hepatocellular carcinoma, the latest clinical trials have also confirmed that traditional Chinese medicine can help reduce its incidence and improve the prognosis in patients [5,6]. Practitioners of traditional Tibetan medicine (TTM), an influential part of Chinese herbal medicine, have constructed a whole theoretical, clinical medicine system based on empirical experience gained during the past few centuries [7]. TTM is now widely accepted in various Asian countries, and studies have shown that it has definite curative effects in preventing cancerous lesions and reducing adverse reactions after anticancer therapy [8,9,10,11]. Additionally, the complex mechanisms through which TTM therapy functions in various types of cancer have been elucidated using emerging network pharmacology-based methods [12,13]. Since network pharmacology-based techniques conform to the “multi-target” and “multi-pathway” concepts of TTM treatment, they can provide researchers with a systematic understanding of the interacting networks of disease targets, medications, and pathways, which is critical in TTM-based drug development [14].

As a widely accepted TTM drug, Liuwei Muxiang (LWMX) pills have been recorded in the officially promulgated *TTM Standard* by the Chinese Ministry of Health since 1995 (drug code: WS3-BC-0283-95). The prescription of LWMX pills consists of six traditional Chinese herbal drugs, including *Aucklandiae Radix* (Chinese: “Muxiang”), *Phyllanthi Fructus* (Chinese: “Yuganzi”), *Amomi Fructus Rotundus* (Chinese: “Doukou”), *Piperis Longi Fructus* (Chinese: “Biba”), *Punica granatum* (Chinese: “Shiliuzi”), and *Veronica eriogyne* (Chinese: “Baxiaga”). According to TTM theory, LWMX pills function by “smoothing blood flow, clearing stomach fire, and removing blood stasis”, which means that LWMX pills have anti-inflammatory and anticancer effects, especially in gastrointestinal diseases. Several previous studies have reported LWMX pills’ effectiveness and potential mechanisms in treating gastrointestinal inflammation and carcinoma [15,16,17]. Nevertheless, the mechanism through which LWMX pills function in CRC remains unexplored.

In this current study, we utilized network pharmacology-based analysis to identify the pharmacological interaction network of LWMX pills with CRC. Furthermore, several machine learning methods were employed to identify the target hub genes related to the anti-CRC effects of LWMX pills. Subsequently, we performed further validation with immune infiltration and molecular docking analyses. Our findings demonstrate the potential anti-CRC mechanism of LWMX pills at the molecular and pathway levels, providing novel evidence for TTM application in CRC treatment.

## 2. Results

### 2.1. Acquisition of the Drug Targets and Disease Targets

**Figure 1:**
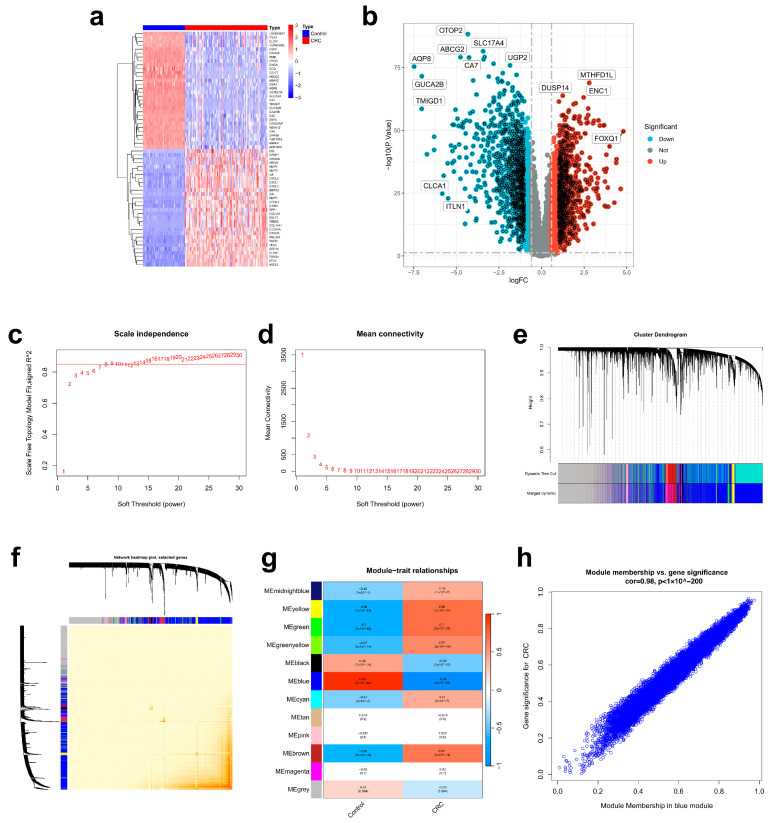
Acquisition of relevant targets of LWMX pills in CRC. (**a**) Heat map of the 50 most significant DEGs. (**b**) Volcano plot of the DEGs. (**c**,**d**) Scale independence and mean connectivity analysis in WGCNA. (**e**) Cluster dendrogram and separation of gene modules in WGCNA. Different modules are represented using different colors. (**f**) Network heat map of the correlation among the module genes. (**g**) The diagram of module–trait relationship analysis for the 12 modules. (**h**) The scatterplot of GS for CRC vs. MM in the blue module.

By searching the Traditional Chinese Medicine Systems Pharmacology Database and Analysis Platform (TCMSP) and the SwissTargetPrediction database, we obtained 81 active ingredients from the 6 TTM constituents of LWMX pills, which correlated with 614 target genes (Supplementary Tables S1 and S2). Before performing differential expression analysis, we normalized the CRC dataset to eliminate the batch effect (Supplementary Figure S1). By performing differential expression analysis on the GSE44076 dataset, we discovered 1877 differentially expressed genes (DEGs). We graphically represent our data with a heat map and a volcano plot (Figure 1a,b; Supplementary Table S2). Furthermore, we employed weighted gene correlation network analysis (WGCNA) to establish a gene coexpression network using the GSE44076 dataset to identify key gene modules related to CRC. According to the scale independence and mean connectivity analyses, we found that the optimal soft threshold was eight (Figure 1c,d). Subsequently, we constructed a coexpression network, clustered the genes, and divided the gene modules using the dynamic tree cut method. After merging similar modules, we obtained 12 different gene modules, and the cluster dendrogram is depicted in Figure 1e. Moreover, based on the gene coexpression network, we plotted a network heat map of the correlations among genes in each module (Figure 1f). Afterwards, the module–trait relationship analysis of these 12 modules was performed, and the results suggested that the blue module (i.e., MEblue) showed the most prominent association with CRC/non-CRC control phenotypes (Figure 1g). Further analysis revealed that there was a significant positive correlation between gene significance (GS) for CRC and module membership (MM) in the blue module (Figure 1h). In summary, the 9534 genes in the blue module were considered potential target genes for CRC (Supplementary Table S2).

### 2.2. Potential Target Prediction of LWMX Pills in CRC and Construction of Protein–Protein Interaction (PPI) and Drug–Disease Networks

**Figure 2:**
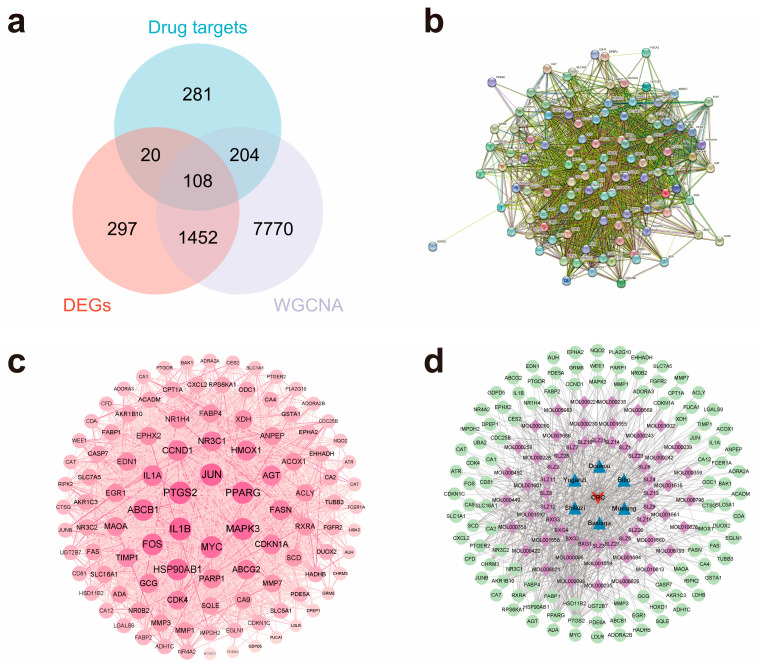
Potential target prediction of LWMX pills in CRC treatment and construction of PPI and drug–disease network. (**a**) Venn diagram of potential target prediction. (**b**) The original PPI network of the intersecting targets. (**c**) The visualized PPI network of the intersecting targets. (**d**) LWMX pill–CRC network diagram. The red arrow represents CRC. The blue triangles represent LWMX pills’ constituents. The purple squares represent active ingredients. The green circles represent the intersecting targets. The connecting lines represent the associations among nodes.

We obtained 108 genes by computing the intersection of the DEGs, the key module genes in the WGCNA, and the target genes of the active ingredients in the LWMX pills (Figure 2a; Supplementary Table S2). These intersecting genes were predicted to be potential targets of LWMX pills in CRC treatment. By importing these intersecting target genes into the Search Tool for the Retrieval of Interacting Genes/Proteins (STRING) database, we obtained a primary PPI network diagram (Figure 2b). Afterward, we input the original PPI information into Cytoscape 3.9.0 for visualization processing (Figure 2c). In the resulting visualized PPI network, the higher the degree value of a given target protein, the larger its node size, indicating that the protein played a more critical role in CRC treatment using LWMX pills by interacting with other targets. The top 20 target proteins with the highest degree values and their PPI information are shown in Table 1. Furthermore, we constructed a LWMX pill–CRC network, including 60 active ingredients, 108 intersecting target genes, 6 TTM constituents, and 1 CRC disease node (Figure 2d). The active ingredients with the highest degree values, such as quercetin, (-)-epigallocatechin-3-gallate, luteolin, palmitoleic acid, and oleic acid, might interfere with the development of CRC through interactions with cancer-related targets (Table 2).

### 2.3. Functional Enrichment Analysis and Construction of Ingredient–Target–Pathway Network

**Figure 3:**
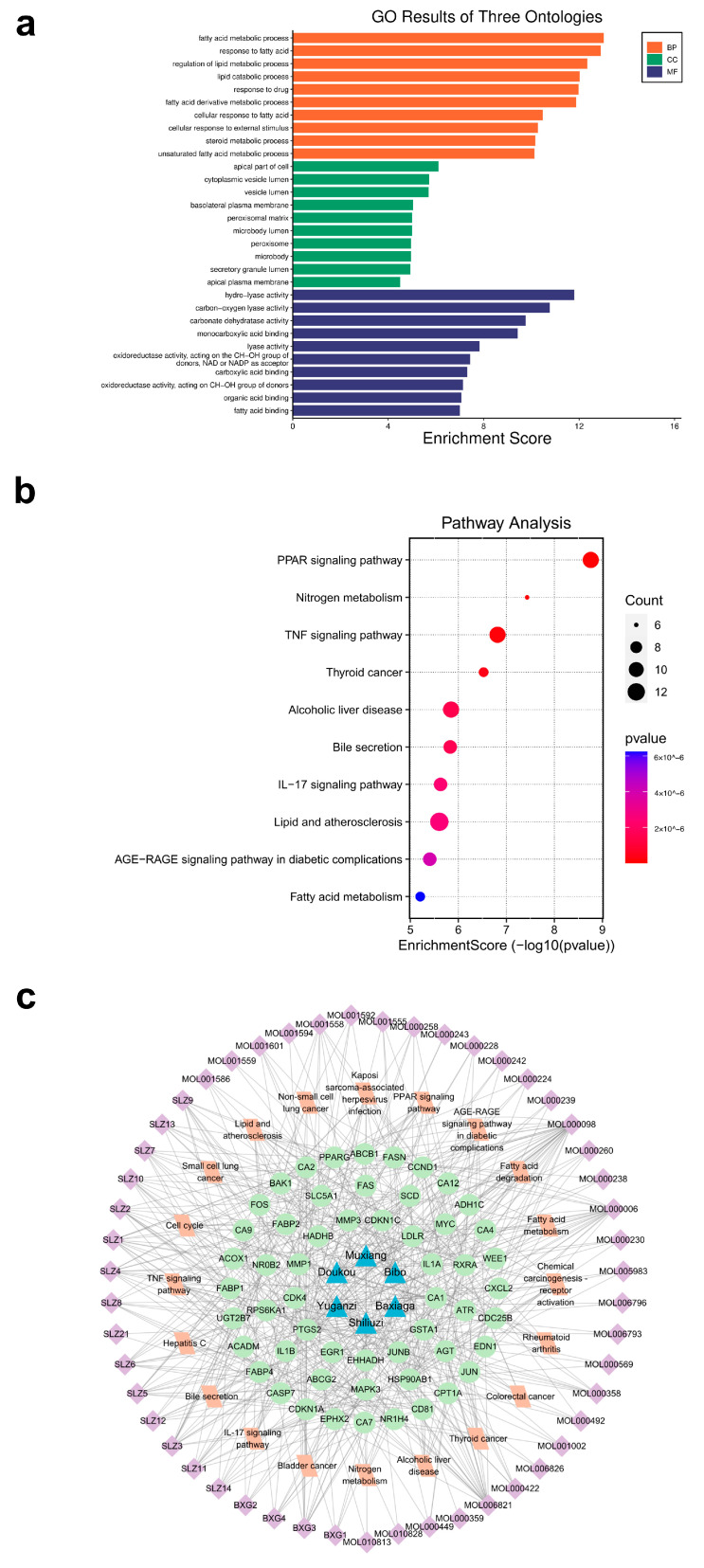
Functional enrichment analysis and the ingredient–target–pathway network of LWMX pills in CRC. (**a**) Bar plot from the GO analysis. (**b**) Bubble plot from the KEGG analysis. (**c**) The ingredient–target–pathway network diagram in CRC treatment using LWMX pills. The blue triangles represent LWMX pills’ constituents. The green circles represent the intersecting targets. The orange rectangles represent signaling pathways. The purple squares represent active ingredients. The connecting lines represent the associations among nodes.

We performed Gene Ontology (GO) and Kyoto Encyclopedia of Genes and Genomes (KEGG) pathway enrichment analyses on these 108 intersecting target genes. The results of the GO analysis are shown in Figure 3a. The potential target genes were mainly enriched in the following biological process (BP) terms: fatty acid metabolic process, response to fatty acid, regulation of lipid metabolic process, lipid catabolic process, and response to drug. The enriched cellular component (CC) entries included the apical part of cell, cytoplasmic vesicle lumen, vesicle lumen, basolateral plasma membrane, and peroxisomal matrix. The enriched molecular function (MF) terms included hydrolyase activity, carbon-oxygen lyase activity, carbonate dehydratase activity, monocarboxylic acid binding, and lyase activity. In addition, according to the KEGG pathway enrichment analysis, these intersecting target genes were primarily enriched in signaling pathways such as the PPAR signaling pathway, pathways in nitrogen metabolism, the TNF signaling pathway, pathways associated with thyroid cancer, and pathways in alcoholic liver disease (Figure 3b). Furthermore, based on the top 20 pathways in the KEGG analysis, we used Cytoscape 3.9.0 to construct an ingredient–target–pathway network diagram for CRC treatment with LWMX pills (Figure 3c).

### 2.4. Determination of Target Hub Genes with Machine Learning

**Figure 4:**
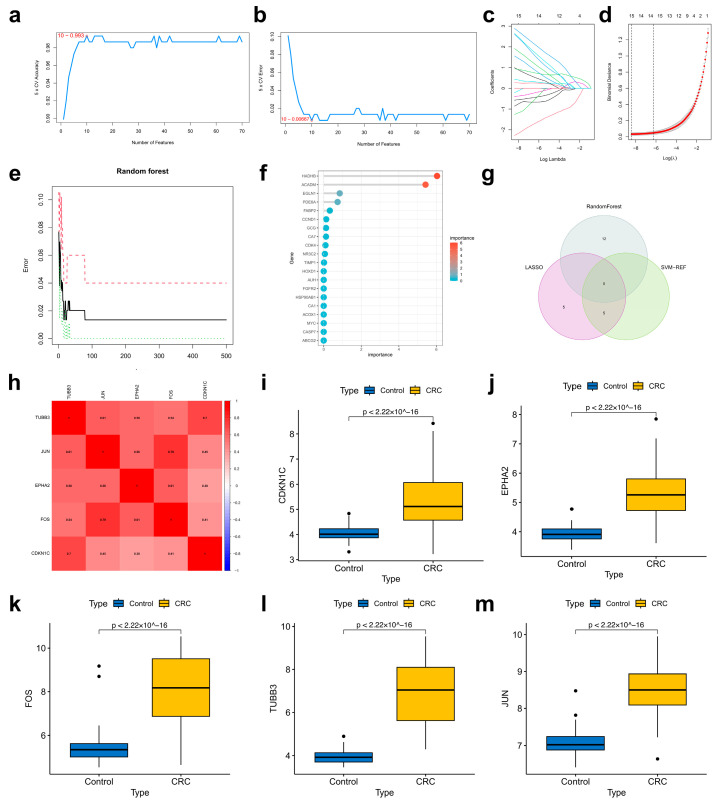
Determining target hub genes through machine learning methods. (**a**,**b**) The accuracy and error rate curves of 5-fold cross-validation based on the SVM-RFE algorithm. (**c**,**d**) The coefficients and regularization diagrams from the LASSO analysis. (**e**,**f**) The error rate curve and VIP evaluation from the RF method. (**g**) Venn diagram of hub target identification. (**h**) Heat map of the correlation analysis among these five hub genes. (**i**–**m**) The box plots of the expression analysis of the hub targets based on the GSE44076 dataset.

To further determine the critical hub genes in CRC treatment using LWMX pills, we set the capability to discriminate between CRC samples and non-CRC samples in the GSE44076 dataset as the evaluation criterion and filtered the 108 intersecting target genes using three machine learning algorithms. With the support vector machine–recursive feature elimination (SVM-RFE) algorithm, we identified 10 core target genes, including *LDLR*, *TUBB3*, *JUN*, *CTSG*, *EPHA2*, *CDKN1C*, *AGT*, *FOS*, *CAT*, and *NQO2* (Figure 4a,b). As a result of least absolute shrinkage and selection operator (LASSO) analysis, 15 genes (*GCG*, *AUH*, *UBA2*, *TUBB3*, *JUN*, *CAT*, *CFD*, *AGT*, *LDLR*, *NQO2*, *GSTA1*, *EPHA2*, *FOS*, *CTSG*, and *CDKN1C*) out of 108 were selected as core target genes (Figure 4c,d). According to the random forest (RF) algorithm, used to calculate the variable importance (VIP) values of all potential target genes, we obtained 17 core genes (*EGR1*, *FOS*, *HADHB*, *ACADM*, *NR4A2*, *JUNB*, *JUN*, *ACLY*, *CDKN1C*, *PTGS2*, *ADORA3*, *EPHA2*, *EGLN1*, *AKR1C3*, *PDE6A*, *TUBB3*, and *CHRM3*) whose VIP values were greater than 0.5 (Figure 4e,f). By computing the intersection of these machine-learning-predicted core target genes, five of them (*TUBB3*, *JUN*, *EPHA2*, *FOS*, and *CDKN1C*) were identified as the target hub genes of CRC treatment with LWMX pills (Figure 4g). Subsequently, we conducted gene correlation (Figure 4h) and expression analyses on different samples for these five target hub genes (Figure 4i–m). The results showed that they are closely related to each other, and their expression levels are significantly higher in CRC tissues than in non-CRC tissues.

### 2.5. Immune Infiltration Analysis

**Figure 5:**
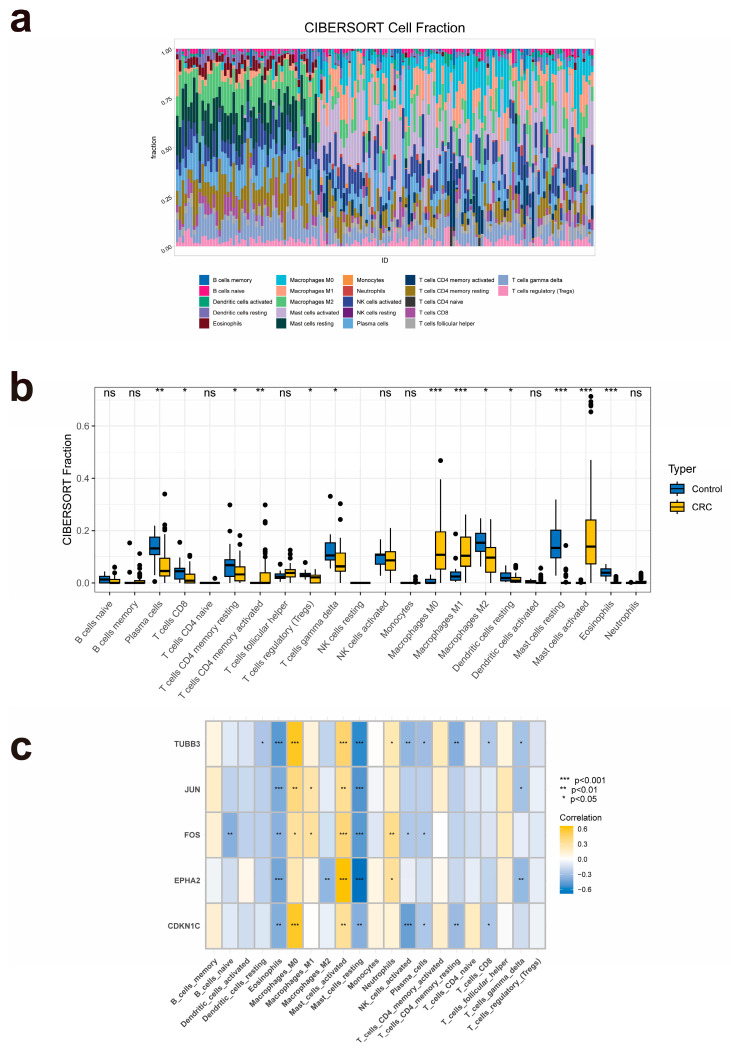
Immune infiltration analysis of the target hub genes. (**a**) Stacked column chart of the infiltration of multiple types of immune cells in each sample of the GSE44076 dataset. (**b**) Box plot of different immune cells’ infiltration between CRC and normal samples. (**c**) Heat map of the correlation between immune infiltration and the hub genes’ expression.

Firstly, we used cell-type identification by estimating relative subsets of RNA transcripts (CIBERSORT) tool to visualize the infiltration of the 22 immune cells within each sample in the GSE44076 dataset (Figure 5a). Then, we analyzed the distinctions in the infiltration of the different immune cells in CRC tissues and non-CRC controls (Figure 5b). The results suggested that in the CRC samples, the proportions of M0 macrophages, M1 macrophages, and activated mast cells were remarkably higher in CRC samples, while those of plasma cells, regulatory T cells, M2 macrophages, resting dendritic cells, resting mast cells, and eosinophils were significantly lower in CRC tissues. Furthermore, we analyzed the correlation between the expression levels of the five target hub genes and the infiltration of various types of immune cells (Figure 5c). The outcomes revealed that the relative abundance of eosinophils and resting mast cells were significantly negatively correlated with the expression levels of all the hub genes; on the other hand, the proportion of activated mast cells was significantly positively associated with the expression levels of all five hub genes.

### 2.6. Molecular Docking

**Figure 6:**
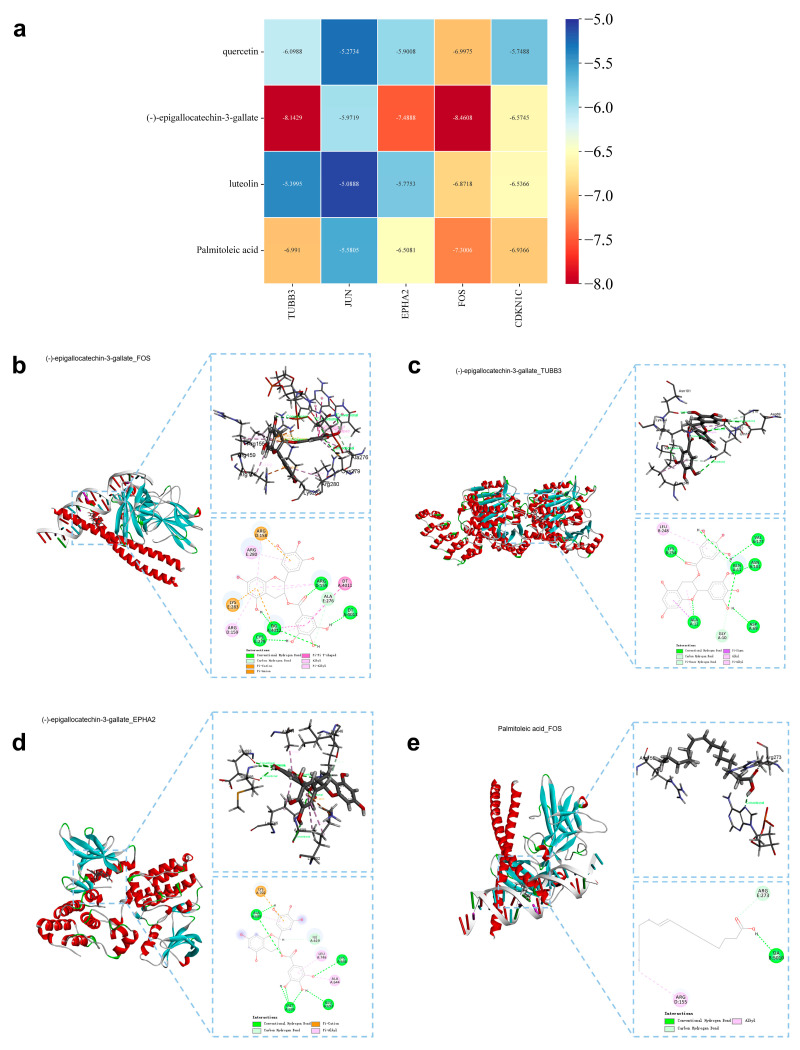
Molecular docking analysis of the active ingredients of LWMX pills with the target hub proteins. (**a**) Heat map of binding energy between active ingredients and target hub proteins (kcal/mol). (**b**) The docking map of (-)-epigallocatechin-3-gallate-FOS interaction, with a binding energy of −8.46 kcal/mol. (**c**) The docking map of (-)-epigallocatechin-3-gallate-TUBB3 interaction, with a binding energy of −8.14 kcal/mol. (**d**) The docking map of (-)-epigallocatechin-3-gallate-EPHA2 interaction, with a binding energy of −7.49 kcal/mol. (**e**) The docking map of palmitoleic acid-FOS interaction, with a binding energy of −7.30 kcal/mol.

Molecular docking analysis was conducted to verify the binding potential between the active ingredients of LWMX pills and the target hub genes. The corresponding structural files of the five target hub genes from the Protein Data Bank (PDB) were prepared as protein receptors, including TUBB3 (PDB ID: 6S8L), JUN (PDB ID: 6Y3V), EPHA2 (PDB ID: 1MQB), FOS (PDB ID: 1A02), and CDKN1C (PDB ID: 4G5Y). The four active ingredients with the highest degree values were selected as drug ligands: quercetin, (-)-epigallocatechin-3-gallate, luteolin, and palmitoleic acid. The heat map of target–ingredient binding energy is shown in Figure 6a. It is widely accepted that a binding energy between a receptor protein and a ligand compound of lower than −5 kcal/mol indicates corroborative binding affinity [18]. Our findings revealed that all four active ingredients had remarkable binding potential with respect to the target hub genes. Moreover, several typical target–ingredient binding interactions with intense binding activity are visualized in Figure 6b–e.

## 3. Discussion

CRC has become one of the most prevalent malignancies, imposing a severe public health burden worldwide. It is the third most common carcinoma and the second most fatal malignant cancer [1]. Although surgical resection, chemoradiotherapy, and immunotherapy are applied for CRC treatment in clinical practice, their effectiveness is frequently limited by poor survival improvement, along with drug resistance, unbearable side effects, and high costs [3,19]. Consequently, it is crucial to develop novel potential drugs for CRC treatment with higher effectiveness and lower toxicity.

TTM has been employed to treat various types of cancer for centuries and is generally accepted across China and several other Asian countries [8]. It has been demonstrated that TTM drugs are efficacious against various types of cancers: they prevent inflammatory injuries and have comparatively weaker side effects [20,21]. As a conventional anti-inflammation and anticancer TTM drug, LWMX pills comprise six Tibetan herbal medications that have anti-CRC potential. Specifically, numerous studies have indicated that *Punica granatum* exhibits strong anti-CRC therapeutic ability [22,23,24]. Additionally, *Phyllanthi Fructus* has been reported to have the potential to protect normal human colon epithelial NCM460 cells from mitotic aberrations and genomic instability partially by regulating the spindle assembly checkpoint [25]. In addition, it was suggested that a herbal preparation of *Aucklandiae Radix* can effectively ameliorate 5-FU-induced gastrointestinal mucositis in CRC chemotherapy [26]. Notably, a recent mouse-model-based study by Dhondrup et al. proved that LWMX pills are able to treat chronic gastritis and prevent gastric cancer progression by inhibiting inflammation and oxidative stress [15]. Despite TTM practitioners commonly prescribing LWMX pills for anti-CRC therapy, the molecular mechanism of this medication had not yet been fully elucidated.

Our study is based on network pharmacology prediction and molecular docking analysis, since these methods are suitable for comprehensively discovering the interaction network of CRC-related targets, the active ingredients of LWMX pills, and key signaling pathways. Similar techniques have also been utilized in several recent studies on Chinese herbal medicine used for CRC treatment [27,28,29]. In this research, we first identified 81 bioactive ingredients in LWMX pills with 614 drug targets from LWMX pills, 1877 differentially expressed CRC genes, and 9534 coexpression CRC module genes. Next, the resulting 108 intersecting genes were analyzed using the PPI method, GO analysis, and KEGG enrichment analysis. LWMX pill–CRC interaction networks and the outcomes of the functional enrichment analysis indicated that multitarget CRC treatment with LWMX pills involves multiple biological processes, such as inflammatory responses and cell cycle regulation. Subsequently, five target hub genes (*TUBB3*, *JUN*, *EPHA2*, *FOS*, and *CDKN1C*) were identified by using several machine learning algorithms. The findings of the immune infiltration analysis suggested that LWMX pills can affect the CRC immune microenvironment by regulating the expression levels of target hub genes, thereby exerting a therapeutic effect on CRC. Finally, the molecular docking results revealed stable binding affinity between all target hub proteins and the primary active ingredients of LWMX pills; therefore, we speculate that these possible interactions between the two are essential in the CRC regulation mechanism of this TTM medication.

Following the evaluation of the diagnostic potential of the intersecting genes, we selected five target hub genes using machine learning methods. One of them, TUBB3, was found to have a positive correlation with epithelial–mesenchymal transition, cell growth, and apoptosis in several CRC cell lines [30,31]. As a crucial part of the CRC-related JNK signaling pathway, JUN can be regulated by numerous upstream targets to further impact tumor growth, CRC cell invasion, and apoptosis [32,33]. In accordance with the results of our KEGG analysis, Yan et al. recently found that in the IL-17 pathway, the deletion of the IL-17 receptor decreases the expression level of A20, which activates the JNK/c-JUN pathway and promotes tumor invasion, growth, and metastasis in patients with CRC [34]. High serum levels of EPHA2 have been previously determined in patients with CRC [35]. Additionally, the methylation of EPHA2 is regulated by several N6-methyladenosine modification proteins, thus promoting vasculogenic mimicry formation via PI3K/AKT/mTOR and ERK signaling pathways in CRC [36]. The transcription level of FOS has been shown to be downregulated by piperlongumine, an active ingredient of *Piperis Longi Fructus*, which can further inhibit cell growth, colony formation, and in vivo tumorigenesis in CRC [37]. CDKN1C has been reported to play a role in the regulation of the CRC cell cycle and drug resistance to paclitaxel [38]. In a study by Yang et al., CDKN1C was proven to participate in regulating the CRC cell cycle and proliferation under the influence of Lappaol F, a natural compound from a Chinese herbal drug [39].

By performing an immune infiltration analysis of the five target hub genes, we found that they likely play a critical role in the invasion process of various immune cell types, especially mast cells and eosinophils. It is widely accepted that the CRC-infiltrating eosinophils mainly regulate their antitumor cytotoxicity by regulating cytokines such as IFNγ and IL-18 [40,41]. However, the function of the CRC-infiltrating mast cells remains controversial [42]. In addition, according to our molecular docking outcomes, (-)-epigallocatechin-3-gallate seems to be a critical bioactive ingredient in LWMX pills. In terms of antigastrointestinal cancer therapy, this compound has been reported to be involved in the suppression of tumor cell proliferation, inhibition of metastasis, induction of cell cycle arrest, prevention of inflammation process, and blockage of tumor angiogenesis [43].

There are several limitations to our study. Firstly, more ingredient–target pairs should be analyzed using molecular docking. Secondly, an additional analysis of CRC prognosis using relevant public databases could be considered. Thirdly, our findings need to be further validated with both in vivo and in vitro experiments.

## 4. Materials and Methods

### 4.1. Acquisition of Relevant Targets of LWMX Pills

We first retrieved relevant data about four constituents of LWMX pills, namely, *Aucklandiae Radix*, *Phyllanthi Fructus*, *Amomi Fructus Rotundus*, and *Piperis Longi Fructus*, from the TCMSP (https://www.tcmsp-e.com/, accessed on 10 November 2023) [44]. According to the pharmacokinetic parameters provided by the TCMSP, we determined the qualified active ingredients in these herbal constituents by using filter criteria: both oral bioavailability ≥ 30% and drug likeness ≥ 0.18 [45]. Then, we searched for the corresponding target proteins of each active ingredient. To determine the qualified active ingredients of *Punica granatum* and *Veronica eriogyne*, which were not included in the TCMSP, we additionally reviewed the literature and obtained the typical SMILES structures of the small-molecule monomers mentioned in those studies from the PubChem database (https://pubchem.ncbi.nlm.nih.gov/, accessed on 10 November 2023) [46,47]. Afterward, we imported these SMILES files into the SwissTargetPrediction database (http://www.swisstargetprediction.ch/, accessed on 10 November 2023) to predict the corresponding target proteins for these bioactive compounds. These genes’ UniprotIDs were then interpreted using the ID mapping tool of the Uniprot database (https://www.uniprot.org/, accessed on 10 November 2023) [48]. At last, we combined the targets’ information mentioned above and obtained the drug targets of LWMX pills after removing any duplicates.

### 4.2. Acquisition of CRC-Related Targets

We searched the CRC dataset from the Gene Expression Omnibus (GEO) database (http://www.ncbi.nlm.nih.gov/geo/, accessed on 10 November 2023) using the keyword “Colorectal cancer”. As the training dataset obtained from our retrieval, the GSE44076 dataset contained data from 98 CRC samples and 50 healthy control samples [49]. All the data analyzed in this study were extracted from the GEO database; thus, no ethical approval or informed consent was required. In the next step, we used R 4.1.2 to normalize the above data and then identified the DEGs associated with CRC using two criteria: |log_2_ fold change (FC)| ≥ 0.585 and adjusted *p*-value < 0.05. Subsequently, we performed WGCNA to determine coexpression modules [50]. The top 25% most significant DEGs were applied in the WGCNA to ensure the accuracy of the results. Firstly, we selected an optimal soft threshold to construct a weighted adjacency matrix and further converted it into a topological overlap matrix (TOM). When the minimum module size was set to 100, modules were created using the TOM similarity metric using the hierarchical clustering tree algorithm. Each module is represented by a certain color. The relationship between modules and disease states was quantified according to MM. GS was defined as the correlation between a gene and the corresponding clinical phenotype.

### 4.3. Potential Target Prediction of LWMX Pills in CRC Treatment

By using a Venn diagram drawn with R 4.1.2, we intersected those previously described DEGs, the CRC-associated targets that were discovered with WGCNA, and the drug targets of LWMX pills to obtain several intersecting target genes that were predicted to be potential targets of LWMX pills in CRC treatment.

### 4.4. Construction of PPI Network and Drug–Disease Network

We imported the aforementioned intersecting targets into the STRING database (https://cn.string-db.org/, accessed on 14 November 2023), an online bioinformatics database designed to provide information on gene and protein interactions, in order to obtain a PPI network in the context of LWMX pill–CRC interactions [51]. Furthermore, we selected interaction scores ≥ 0.4 as a criterion to obtain a simplified PPI network diagram. Subsequently, we adopted Cytoscape 3.9.0 to visualize the PPI diagram and constructed the LWMX pill–CRC network diagram [52].

### 4.5. Functional Enrichment Analysis and Construction of Ingredient–Target–Pathway Network

To explore the possible biological functions and main signaling pathways in CRC treatment with LWMX pills, we conducted GO and KEGG pathway enrichment analyses on the intersecting targets using the ClusterProfiler package in R software 4.1.2 [53,54]. After filtering with the criterion of q-value <0.05, we ranked the qualifying terms in descending order according to their enrichment scores. The bar plot and bubble plot of our data were thus created. In addition, based on the LWMX pill–CRC network and the outcomes of the functional enrichment analysis, an ingredient–target–pathway network diagram of CRC treatment with LWMX pills was delineated using Cytoscape 3.9.0 (Institute for Systems Biology, Seattle, WA, USA).

### 4.6. Determination of Hub Genes with Machine Learning

Three machine learning algorithms, LASSO, SVM-RFE, and RF, were used to further identify the hub genes among the intersecting target genes [55,56,57]. With these machine learning methods, specific genes that could allow for distinguishing patients with CRC from healthy control subjects were identified among the intersecting targets. The specific targets that were selected by all three algorithms were deemed to be the hub genes in CRC treatment with LWMX pills. In the LASSO analysis, the glmnet package from R 4.1.2 was applied for the 10-fold cross-validation. In the SVM-RFE analysis, the e1071 and svmRadial packages were employed for feature classification. Furthermore, the randomForest package was used to establish an RF classification model, and feature genes were sorted according to their VIP values. Finally, we determined the hub genes by computing the intersection of the genes identified with the machine learning algorithms in the LWMX pill–CRC interaction, which was followed by gene correlation analysis and differential expression analysis.

### 4.7. Immune Infiltration Analysis

We utilized the CIBERSORT algorithm (https://cibersortx.stanford.edu/, accessed on 15 November 2023) for immune infiltration analysis, with the aim of evaluating the potential link between the target hub genes and changes in the immune microenvironment of patients with CRC [58]. After estimating the relative proportion of 22 types of immune cells in each sample from the GSE44076 dataset, the immunological scores of these samples were calculated using the ESTIMATE algorithm. In addition, the correlation between the hub genes and the immune cells was determined by performing Spearman correlation analysis.

### 4.8. Molecular Docking

At first, we extracted the target hub proteins’ structures from the PDB database (https://www.rcsb.org/, accessed on 17 November 2023) and acquired the identified active ingredients’ structures from the PubChem database [59]. These structural files were then preprocessed with Auto Dock 4.2.6 (Scripps Research, La Jolla, CA, USA) (i.e., we removed water and added hydrogen) and converted them into PDBQT format [60]. Next, the binding sites of the target hub proteins were analyzed, and the corresponding docking active pockets were determined. After importing the structural files of the proteins and vital active ingredients into Auto Dock Vina 1.1.2 (Scripps Research, La Jolla, CA, USA), we performed the docking verification. Subsequently, the output results were plotted into a heat map to visualize the potential binding affinity of these crucial bioactive compounds and the target hub proteins. Finally, several emblematic docking maps were depicted using Discovery Studio Visualizer v2021 (BIOVIA, Paris, France).

## 5. Conclusions

In this study, we identified the vital active ingredients and potential targets of LWMX pills in CRC treatment. LWMX pill–CRC interaction networks and the outcomes of the functional enrichment analysis indicated that multitarget CRC treatment using LWMX pills involves multiple biological processes, such as the inflammatory response and cell cycle regulation. We identified five target hub genes (TUBB3, JUN, EPHA2, FOS, and CDKN1C) using machine learning tools. The findings of the immune infiltration analysis suggested that LWMX pills can affect the immune microenvironment in CRC by regulating the expression levels of the target hub genes. The molecular docking outcomes revealed promising binding affinity among all target hub proteins and the primary active ingredients of LWMX pills, such as quercetin, (-)-epigallocatechin-3-gallate, luteolin, and palmitoleic acid. Our findings illustrate the anti-CRC potential and mechanisms of LWMX pills at the molecular and pathway levels and provide novel insights into multitarget medication for CRC treatment.

## Figures and Tables

**Table 1:** Information on the top 20 targets sorted by degree value.

Full Target Name	Target Acronym	Degree	Betweenness Centrality	Closeness Centrality
Interleukin-1 beta	IL1B	90	0.035500531	0.868852459
Prostaglandin G/H synthase 2	PTGS2	89	0.035478698	0.861788618
Transcription factor Jun	JUN	89	0.035418858	0.861788618
MAP kinase-activated protein kinase 3	MAPK3	85	0.030411643	0.834645669
Peroxisome proliferator-activated receptor gamma	PPARG	85	0.026629396	0.834645669
Myc proto-oncogene protein	MYC	83	0.026441028	0.821705426
Heat shock protein HSP 90-beta	HSP90AB1	82	0.026165911	0.815384615
Protein c-Fos	FOS	80	0.034046611	0.796992481
ATP-dependent translocase ABCB1	ABCB1	79	0.025695306	0.796992481
Interleukin-1 alpha	IL1A	72	0.016995181	0.757142857
G1/S-specific cyclin-D1	CCND1	71	0.015427766	0.75177305
Heme oxygenase 1	HMOX1	67	0.011811346	0.726027397
Glucocorticoid receptor	NR3C1	67	0.02356451	0.731034483
Angiotensinogen	AGT	66	0.014954789	0.721088435
Fatty acid synthase	FASN	65	0.020256702	0.716216216
Cyclin-dependent kinase inhibitor 1	CDKN1A	64	0.017355073	0.716216216
Broad substrate specificity ATP-binding cassette transporter ABCG2	ABCG2	64	0.010097723	0.711409396
Poly [ADP-ribose] polymerase 1	PARP1	60	0.008521068	0.692810458
Cyclin-dependent kinase 4	CDK4	59	0.014661166	0.692810458
Pro-glucagon	GCG	59	0.009433137	0.688311688

**Table 2:** Information on the top 20 active ingredients sorted by the degree value.

Ingredient Code	Ingredient Name	Degree	Betweenness Centrality	Closeness Centrality
MOL000098	Quercetin	46	0.028875395	0.393665158
MOL006821	(-)-Epigallocatechin-3-gallate	30	0.034899926	0.404651163
MOL000006	Luteolin	24	0.009673829	0.373390558
SLZ2	Palmitoleic acid	22	0.011690763	0.397260274
SLZ5	Oleic acid	21	0.010631126	0.395454545
SLZ11	9,12-hexadecadienoic acid	19	0.009826783	0.397260274
SLZ6	Linoleic acid	19	0.008236272	0.391891892
SLZ1	Palmitic acid-13C	19	0.009049947	0.376623377
SLZ9	Gondoic acid	18	0.008683739	0.390134529
SLZ10	Linolenic acid	18	0.008140464	0.390134529
SLZ3	Margaric acid	18	0.007711383	0.375
SLZ7	Γ-linolenic acid	18	0.009737845	0.391891892
BXG3	Protocatechuic acid	18	0.020991044	0.379912664
SLZ4	Stearic acid-1-13C	17	0.00703053	0.373390558
SLZ12	L-aspartic acid	15	0.005323021	0.345238095
SLZ13	L-glutamic acid	15	0.005323021	0.345238095
SLZ14	Sericic acid	14	0.00715489	0.381578947
SLZ8	Arachidic acid	13	0.003773497	0.348
MOL000358	Beta-sitosterol	10	0.004651937	0.335907336
MOL000422	Kaempferol	10	0.003127066	0.341176471

## Data Availability

The dataset (GSE44076) analyzed in this current study is available from the Gene Expression Omnibus database repository (https://www.ncbi.nlm.nih.gov/geo/). Other relevant raw data can be provided upon reasonable request (e.g., for further analysis).

## Supplementary Materials

The following supporting information can be downloaded at: https://www.mdpi.com/article/10.3390/ph17040429/s1. Figure S1: Normalization of the GSE44076 dataset; Table S1: Information of the active ingredients from Liuwei Muxiang pills; Table S2: Information of the Liuwei Muxiang pills’ targets, differentially expressed genes, module genes in WGCNA and the intersecting targets.

## Abbreviations

LWMX pills	Liuwei Muxiang pills
CRC	colorectal cancer
TTM	traditional Tibetan medicine
TCMSP	Traditional Chinese Medicine Systems Pharmacology Database and Analysis Platform
DEGs	differentially expressed genes
WGCNA	weighted gene correlation network analysis
PPI	Protein–protein interaction
STRING	Search Tool for the Retrieval of Interacting Genes/Proteins
GO	Gene Ontology
BP	biological process
CC	cellular component
MF	molecular function
KEGG	Kyoto Encyclopedia of Genes and Genomes
LASSO	least absolute shrinkage and selection operator
SVM-RFE	support vector machine-recursive feature elimination
RF	random forest
VIP	variable importance
GEO	Gene Expression Omnibus
TOM	topological overlap matrix
MM	module membership
GS	gene significance
CIBERSORT	cell-type identification by estimating relative subsets of RNA transcripts
PDB	Protein Data Bank
