# Supplementary material for: Potential Mechanism of Tibetan Medicine Liuwei Muxiang Pills against Colorectal Cancer: Network Pharmacology and Bioinformatics Analyses
Source: Pharmaceuticals (Basel). 2024 Mar 27;17(4):429. doi: 10.3390/ph17040429 (PMC11054834; doi:10.3390/ph17040429)

Supplementary Figure S1 Normalization of the GSE44076 dataset

a The dataset before normalization. **b** The dataset after normalization.

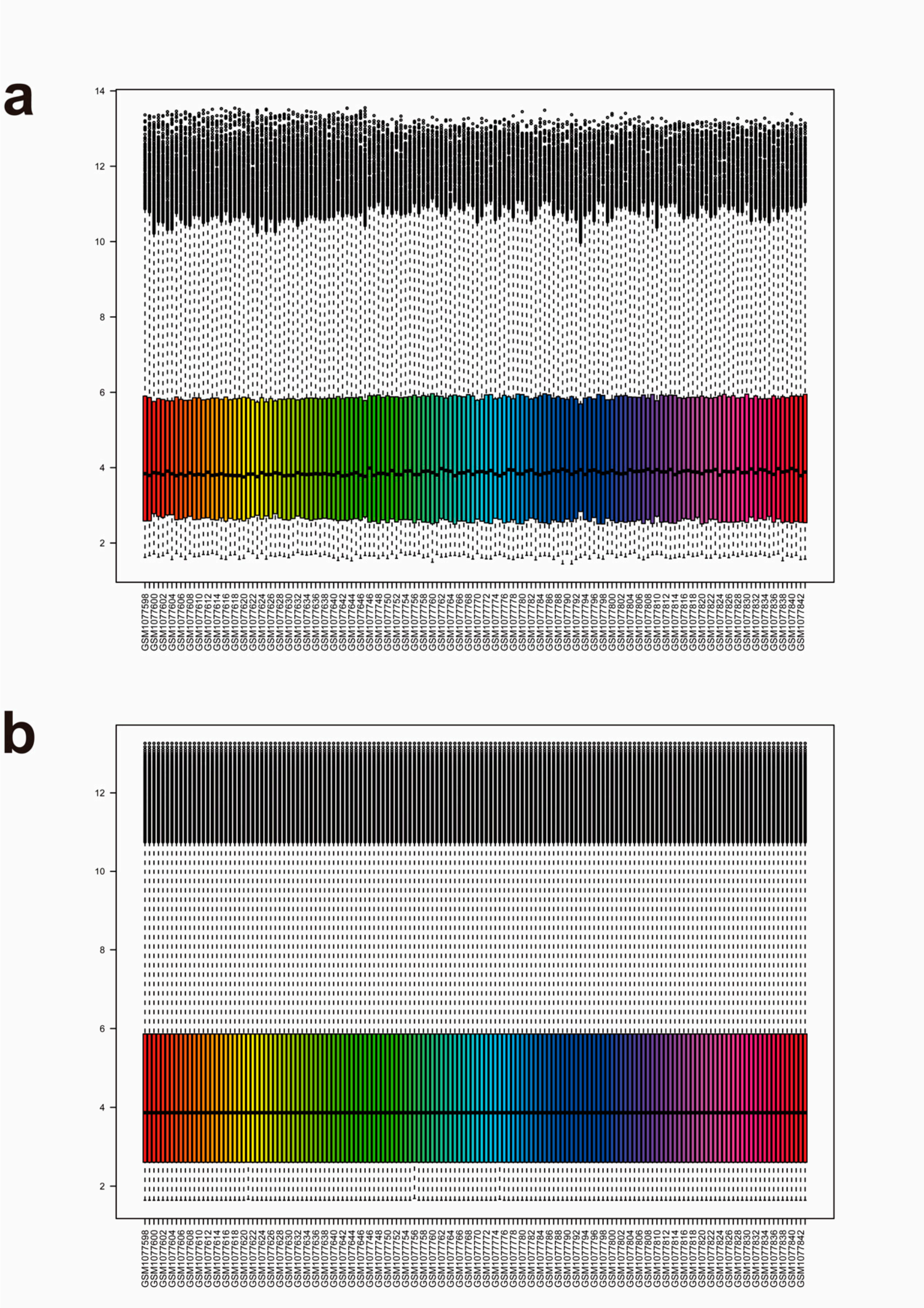

Supplement: Supplementary file 1 [file pharmaceuticals-17-00429-s001.zip › Supplementary Figure S1.pdf]
